# Green Synthesis of Chitosan/Silver Nanoparticles Using *Citrus paradisi* Extract and Its Potential Anti-Cryptosporidiosis Effect

**DOI:** 10.3390/pharmaceutics16070968

**Published:** 2024-07-22

**Authors:** Muslimah N. Alsulami, Eman S. El-Wakil

**Affiliations:** 1 Department of Biology, College of Science, University of Jeddah, Jeddah 21589, Saudi Arabia; mnal-sulami@uj.edu.sa; 2 Department of Parasitology, Theodor Bilharz Research Institute, Kornaish El-Nile, Warrak El-Hadar, Imbaba (P.O. 30), Giza 12411, Egypt

**Keywords:** antiparasitic, *Citrus paradisi* peel, *Cryptosporidium parvum*, chitosan, green synthesis, silver nanoparticles

## Abstract

*Cryptosporidium parvum* (*C. parvum*) is one of the most prevalent species infecting humans and animals. Currently, the only FDA-licensed drug to treat cryptosporidiosis is nitazoxanide (NTZ), with no efficacy in immunocompromised hosts. *Citrus paradisi* (*C. paradisi*) has demonstrated anti-protozoal activities. This study aimed to investigate the anti-cryptosporidiosis effect of *C. paradisi* peel extract, either alone or in mediating the green synthesis of chitosan silver nanoparticles (Cs/Ag NPs), compared to NTZ. Mice were sorted into nine different groups. The effectiveness of the treatments was evaluated using parasitology, histopathology, immunohistochemistry, and immunology. *C. paradisi* outperformed nitazoxanide regarding oocyst shedding (79% vs. 61%). The effectiveness of NTZ Cs/Ag NPs and Citrus Cs/Ag NPs was enhanced to 78% and 91%, respectively. The highest oocyst inhibition was obtained by combining NTZ and Citrus Cs/Ag NPs (96%). NF-κB, TNF-α, and Il-10 levels increased in response to infection and decreased in response to various treatments, with the highest reduction in the group treated with combined NTZ citrus Cs/Ag NPs. Combining *C. paradisi* with NTZ could have a synergistic effect, making it a potentially effective anti-cryptosporidiosis agent. Utilizing *C. paradisi* in the green synthesis of Cs/Ag NPs improves the therapeutic response and can be used to produce novel therapeutic antiparasitic drugs.

## 1. Introduction

*Cryptosporidium parvum* (*C. parvum*) is one of the most common intracellular protozoan parasitic pathogens that can cause humans and animals enteric disease worldwide [1]. The route of infection is through the consumption of infectious oocysts through either contaminated food or drink or the respiratory route [2].

A *C. parvum* infection is a major health concern, since the infectious oocysts are incredibly persistent in the environment and resistant to almost all conventional disinfection methods and water treatments [1].

Infection with *Cryptosporidium* has a variety of disease presentations that could be influenced by the host’s immune status, nutrition, and age. It is self-limited in immunocompetent hosts that either do not display symptoms or display mild symptoms, but life-threatening diarrheal disease can take place in immunodeficient hosts, young children, and neonatal animals [3].

Regarding cryptosporidiosis treatment, the only medication currently licensed by the US Food and Drug Administration (FDA) to treat cryptosporidiosis is nitazoxanide, with moderate efficacy in immunocompetent hosts. More critically, there are currently no effective therapeutic medications available to treat severe cases of cryptosporidiosis in immunocompromised individuals or young infants, and infection could be fatal [4]. Adding to this, there are no vaccines available for cryptosporidiosis [5]. All these factors make the quest for novel therapeutic agents or new modalities to treat cryptosporidiosis, especially in immunodeficient hosts, a pressing need.

The use of medicinal plants in treating parasite diseases including cryptosporidiosis has increased recently, and several plant extracts are the subject of pharmacological studies [6]. Citrus fruits are popular everywhere in the world. One of the economically important plants in this group is the *Citrus paradisi* (*C. paradisi*), or grapefruit, which is well known for its dietary and medicinal properties [7].

Citrus fruit peels are high in flavanones and polymethoxylated flavones, which are uncommon in other plants. The peel of *C. paradisi* contains flavonoids, glycosides, naringin, and hesperidin, which have different biological actions, encompassing anti-inflammatory, anti-mutagenic, antioxidant, and analgesic activities [8]. Moreover, *C. paradisi* has demonstrated anti-protozoal activities, including against *Giardia duodenalis* [9], *Plasmodium berghei* [10], and *Eimeria* infection [11]. The *C. paradisi* anti-helminthic effect was also detected on *Schistosoma hematobium* [12].

Nanotechnology has become an extremely promising subject in recent years, with great potential for fighting infectious diseases [13]. Silver nanoparticles (Ag NPs) represent an essential development in nanotechnology because of their outstanding reliability and limited chemical reactivity compared to other metals. Due to their unique physicochemical characteristics, Ag NPs have drawn much interest in biological applications [14].

Chitosan (Cs) is a widely available, inexpensive, naturally occurring biopolymer that has been given FDA approval. It possesses several biological activities, such as immune-stimulating, anticancer, and antimicrobial effects [15].

Three methods may be used to synthesize nanoparticles: the chemical, physical, and biological methods. Many attempts have been made to develop green synthesis techniques using microorganisms and plant extracts to minimize the usage of hazardous ingredients and the high cost of the physical and chemical techniques required to synthesize nanoparticles. Since plant-mediated synthesis is ecologically friendly, it is considered a reliable and secure technique for synthesizing nanoparticles [16]. Nanoparticles formed using *C. paradisi* peel extract are stable for a very long period because of the interaction formed between the *C. paradisi* peel extract functional groups and NPs [17].

Because of their biocompatibility and biodegradability, chitosan/silver nanocomposites, a type of silver-based nanocomposites, are a rapidly developing class of bio-nanostructured hybrid materials. They are thought to be potent antibacterial and antiparasitic agents with many encouraging outcomes in the eradication of parasitism in the veterinary sector [18].

In the current work, we have attempted to evaluate the potential anti-cryptosporidiosis efficiency of the *C. paradisi* extract-mediated green synthesis of chitosan (Cs)/silver (Ag) nanoparticles (NPs) in a murine model compared to commercially available nitazoxanide therapy.

## 2. Materials and Methods

### 2.1. The Parasite

Oocysts of *cryptosporidium* were obtained from diarrheic calves and were confirmed genetically as *C. parvum* in our earlier research [19]. The oocysts from the diarrheal stool samples were isolated and purified, stored in a solution of 2.5% potassium dichromate, and preserved until required at 4 °C [20].

### 2.2. Experimental Animals and Grouping

Ninety white Albino laboratory-bred mice weighing 20–25 g each and 6–8 weeks old were used in this study. The mice were obtained from the Theodor Bilharz Research Institute (TBRI) biological unit. The animals used in this study were fed a regular pellet diet and had unlimited water access at TBRI Animal House. Mice were sorted into nine groups (ten mice/group):

Group I: healthy model (healthy mice).

Group II: diseased model (mice were infected and not treated).

Group III: infected mice treated with *C. paradisi* peel extract (100 mg/kg body weight given daily for five consecutive days) [9].

Group IV: infected mice treated with nitazoxanide (100 mg/kg body weight daily given for five consecutive days) [21].

Group V: Infected mice that received a combination of *C. paradisi* peel extract and nitazoxanide.

Group VI: infected mice treated with chitosan/silver nanoparticles (Cs/Ag NPs) at a 50 µg/mouse/day dose for five consecutive days [22].

Group VII: infected mice treated with the *C. paradisi* peel extract-mediated green synthesis of Cs/Ag NPs.

Group VIII: infected mice treated with nitazoxanide loaded on Cs/Ag NPs.

Group IX: Infected mice treated with a combination of nitazoxanide and the *C. paradisi* peel extract-mediated green synthesis of Cs/Ag NPs.

#### 2.2.1. Immunosuppression Induction

Two weeks prior to the induction of infection, the immunosuppression of mice was performed by giving dexamethasone sodium phosphate (Dexazone) orally (0.25 mg/g/day) using an esophageal syringe. The animals were then kept on the same dexamethasone dose for the duration of the experiment [23].

#### 2.2.2. Induction of *Cryptosporidium* Infection

Before initiating the infection, the oocysts were washed at least three times and centrifuged in distilled water until they were clear. This was carried out to remove the K_2_Cr_2_O_7_ [24].

After preparing the infective inoculum, the fluid volume of the inoculum per mouse was calculated and the infective oocyst number at the stock concentrated inoculum was detected. Then, the oocysts were given to the mice through oral–gastric gavage. Each mouse (except mice in group 1) was given approximately 10^4^ oocysts of *Cryptosporidium* per mouse [25] (day 0 post-infection).

### 2.3. Preparation of Drugs

#### 2.3.1. Extraction of Citrus Paradisi Peels

*Citrus paradisi* fruits were bought from a local Cairo market. The peels were removed, collected, and dried in the shade away from direct sunlight. The dry powdered peels (550 g) were extracted via maceration using methanol as the extracting solvent (3 × 1 L) at room temperature. Using a rotatory evaporator, the extract was concentrated at 40 °C (Buchi R-300, Flawil, Switzerland). The obtained methanolic extract (103 g) was kept in a safe container until being used [26].

#### 2.3.2. Preparation of Chitosan/Silver Nanoparticles (Cs/Ag NPs)

The chitosan/silver nanoparticles (Cs/Ag NPs) were produced according to [15]. Briefly, trisodium citrate (1% *w*/*v*) was applied to silver nitrate (AgNO_3_), and then the mixture was stirred for 2 h and sonicated at 1.5 kW for 30 min. The chitosan solution was mixed with the prepared silver solution (1% *w*/*v* in 1% acetic acid) and stirred for 12 h.

#### 2.3.3. Green Synthesis of *C. paradisi* Cs/Ag NPs

The green synthesis of Cs/Ag NPs using *C. paradisi* extract was performed following [27]. First, 95 mL of distilled water was mixed with one mM of silver nitrate. *C. paradisi* peel extract (5 mL) was added to the 95 mL silver nitrate solution. The reaction mixture was set in a magnetic stirrer for 3 h at 700 rpm to detect color changes. Then, chitosan (0.25 g) was added to the solution and stirred in a magnetic stirrer for 3 h at 700 rpm.

#### 2.3.4. Preparation of Nitazoxanide Cs/Ag NPs and Nitazoxanide and *C. paradisi* Cs/Ag NPs

The chitosan/silver nanoparticles (Cs/Ag NPs) and *C. paradisi* Cs/Ag NPs were prepared as mentioned before. Nitazoxanide-loaded Cs/Ag NPs and Nitazoxanide *C. paradisi* Cs/Ag NPs were synthesized by adding NTZ (concentration 150 mg) dropwise with constant stirring, followed by sonication.

### 2.4. Characterization of Nanoparticles

A UV-visible spectrophotometer (UV-1800, Shimadzu, Kyoto, Japan) was used, and it has a 1 nm resolution between 200 and 800 nm. The wavelength (nm) is shown on the *X*-axis, while the absorbance is shown on the *Y*-axis. The biocomponents of the *C. paradisi* extract initiated a reduction in silver ions. At 430 nm, the Ag NPs from *C. paradisi* were observed which confirmed the synthesis of nanoparticles.

The presence of functional groups in the Cs/Ag NPs was verified by FT-IR analysis. Transmission spectra from the 400–3500 cm^−1^ range were measured and recorded after buffer subtraction. Using a Perkin Elmer spectrum 100 FTIR spectrometer, this value was acquired, validating the surface structure and structural alterations of modified proteins at different concentrations of Cs/Ag NPs. These Cs/Ag NPs’ morphological form, size, and shape were further assessed utilizing a transmission electron microscope (TEM) with a Zeiss EM 900 instrument (Carl Zeiss, Oberkochen, Germany) model and Image SP software version 1.2.13.29.

### 2.5. Drug Assessment

#### 2.5.1. Parasitological Examination

Following drug administration, infected mice’s feces were collected at 7, 12, 15, and 20 days post-infection (dpi). For parasitological examination, the *Cryptosporidium* oocyst number was counted in the fecal pellets utilizing cold Kinyoun’s acid-fast stain. Fecal samples were taken from every infected mouse. For every mouse group, the mean number of oocysts was determined. One milligram of the fecal pellet was measured and kept in one milliliter of 10% formalin. Centrifugation was performed for 10 min at 500× *g* to concentrate the fecal solution. The oocyst count in one milliliter of the fecal sample was detected by taking 100 µL of fecal sediment, which was dried, stained, and then subjected to an oil immersion lens (×100) examination. A calculation of the average of three counts and multiplication by ten were carried out to find the oocyst number for 1 mL of fecal sample [28]. The oocyst number was displayed per gram of feces [29].

Each drug’s efficacy was determined according to [30] and expressed as a reduction percentage using the formula below:

$$Efficacy\left(\%\right)=100\times \frac{\left(oocyst\;count\;of\;the\;diseased\;model\;group-oocyst\;count\;of\;infected\;treated\;group\right)}{oocyst\;count\;of\;diseased\;model\;group}$$

#### 2.5.2. Histopathological Examination

When the experiment reached its end, mice were euthanized using light anesthesia. Tissue samples from the small intestine were flushed and fixed using 10% neutral buffered formalin for 72 h. After the samples were cut and processed through several ethanol grades, they were cleaned in Xylene, infiltrated, and implanted in Paraplast tissue embedding media. Then, 5 μm thick tissue sections were developed using a rotatory microtome to demonstrate the intestinal wall. The hematoxylin and eosin (H&E) staining method was used to detect morphological changes in tissue sections [31].

#### 2.5.3. Immunohistochemical Examination Assessment of NFkB

Paraffin-embedded tissue sections 5 μm thick were used for immunohistochemical analysis following the manufacturer’s protocol. H_2_O_2_ (0.3%) was used to treat deparaffinized retrieved tissue sections for 20 min. Then, the samples were incubated overnight at 4 °C within anti-NFκB p65 antibody (GTX54672, GeneTex Inc., Irvine, CA, USA—1:100). Using PBS, tissue sections were washed out and incubated for 20 min with a secondary antibody HRP Envision kit (DAKO), Santa Clara, CA, USA, and then washed out and incubated for 15 min with diaminobenzidine (DAB). Afterward, they were PBS-washed, hematoxylin-counterstained, Xylene-dehydrated, cleared, and then covered for microscopic examination.

According to the method adopted from [32], at least six non-overlapping random fields were chosen and scanned from each intestinal sample for detecting the relative area percentage of mucosal immunoexpression levels of NFκB in immunohistochemically stained sections. Histological analysis using the Leica Application module, which was connected to the Full HD microscopic imaging system (Leica Microsystems GmbH, Wetzlar, Germany), was used for all light microscopic examinations and data collection.

#### 2.5.4. Immunological Analysis of Serum Levels of Interleukin 10 and TNF Alpha Cytokines

Blood samples were centrifuged for 5 min at 4500 rpm; serum was stored at −20 °C until analysis. The measurement of levels of IL10 and TNF alpha was performed according to the manufacture instructions for the ELISA assays, which were supplied by (INNOVA BIOTECH Co., Ltd. (Beijing, China), Cat. No. In-Mo 1242) and (INNOVA BIOTECH Co., Ltd., Cat. No. In-Mo 1920), respectively.

### 2.6. Statistical Analysis

The statistical package for social sciences (IBM SPSS Statistics for Windows, version 26, IBM Corp., Armonk, NY, USA) was used for data analysis. Mean ± SE was used to display continuous normally distributed variables. One-way ANOVA followed by Tukey’s post hoc HSD was used to conduct the multi-group analysis. A statistically significant *p*-Value was defined as <0.05, and a highly significant *p*-Value was defined as <0.001.

## 3. Results

### 3.1. Encapsulation Efficiency

The apparent entrapment efficiency (EE) of the amount of nitazoxanide in Nit- CS/Ag Nano Composite and Citrus-CS/Ag Nano Composite was detected via the indirect method. Briefly, a 2 mL aliquot of Nit- CS/Ag NPs was ultra-centrifuged for 15 min at 10,000 rpm at 4 °C using a Beckman OptimaTM Ultracentrifuge (OptimaTM XL, Indianapolis, IN, USA). The free unencapsulated nitazoxanide and Citrus proportion were spectrophotometrically analyzed (Shimadzu, the model UV-1800 PC, Kyoto, Japan) against a blank at 245 and 280 nm. The apparent entrapment efficiency (%EE) was then calculated by using the following equation:

$$\%EE=[\frac{Total\;Nit-CS/Ag\;Nano\;Composite-Free\;nitazoxanide}{Total\;Nit-CS/Ag\;Nano\;Composite}\times 100]=75\%$$

$$\%EE=[\frac{Total\;Citrus\;CS/Ag\;Nano\;Composite-Free\;Citrus}{Total\;Citrus\;CS/Ag\;Nano\;Composite}\times 100]=85\%$$

Due to phytochemical components found in the plant extracts, *C. paradisi* peel extract was used as a green bioactive reducing agent to reduce silver ions into silver nanoparticles. Spectrophotometry analysis was used to track the reaction process.

### 3.2. Characterization of Silver Nanoparticles

#### 3.2.1. Color Change and Visual Observation

The reaction mixture’s color-changing (visual observation) during the time of the reaction is the main indication of nanoparticle synthesis. The change in color from colorless (AgNO_3_ alone) to yellowish is evidence of the formation of the Ag NPs. The color of NTZ Citrus-CS/Ag NPs was a pale-yellow turbid solution due to the chitosan solution. While turbidity decreased, the color intensity increased without nitazoxanide, as seen in Supplementary Materials Figure S1. The reaction medium acquired a colorless solution, which UV-Vis spectrophotometry analysis further confirmed.

#### 3.2.2. UV-Vis Spectroscopy

**Figure 1:**
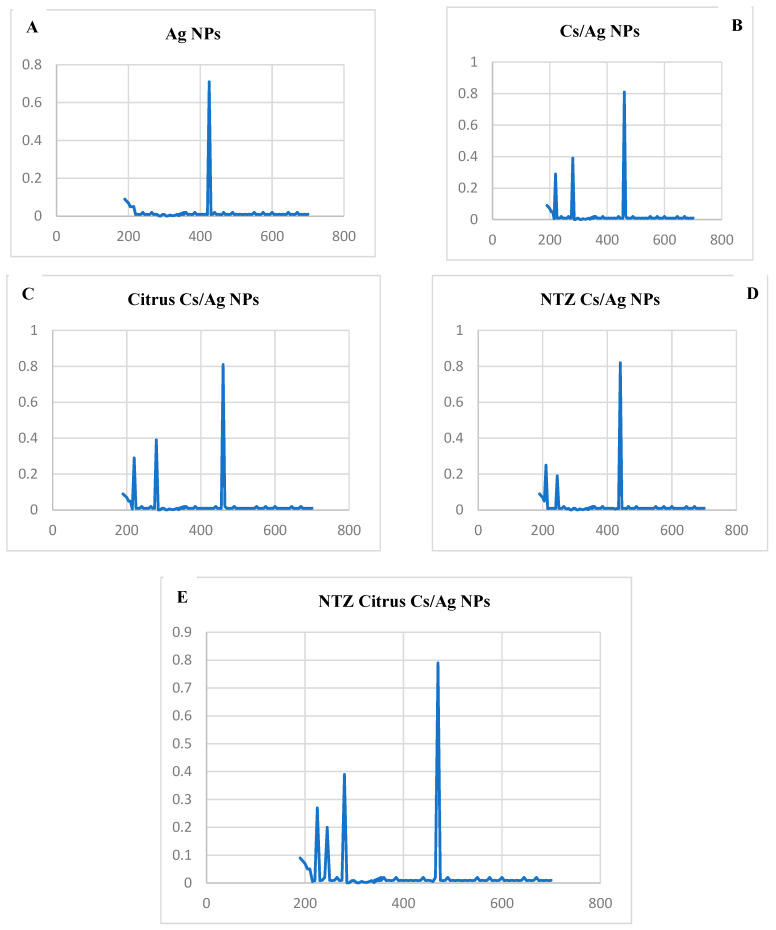
UV-Vis spectroscopy: (**A**) Ag NPs. (**B**) Cs/Ag NPs. (**C**) Citrus Cs/Ag NPs. (**D**) NTZ Cs/Ag NPs. (**E**) NTZ Citrus Cs/Ag NPs.

Sharp peaks in the UV-Vis spectrum in the visible range validate the synthesis of Ag-NPs, which are identified by the visual observation of color change. Analyses using UV–Vis spectrophotometer were performed in the range from 200 to 800 nm in the absorbance spectrum for Ag NPs. A band at 430 nm was seen in the UV-visible absorption spectra of silver nanoparticles made with *C. paradisi*, signifying the presence of spherical silver nanoparticles, as shown in Figure 1.

#### 3.2.3. Transmission Electron Microscopy (TEM)

**Figure 2:**
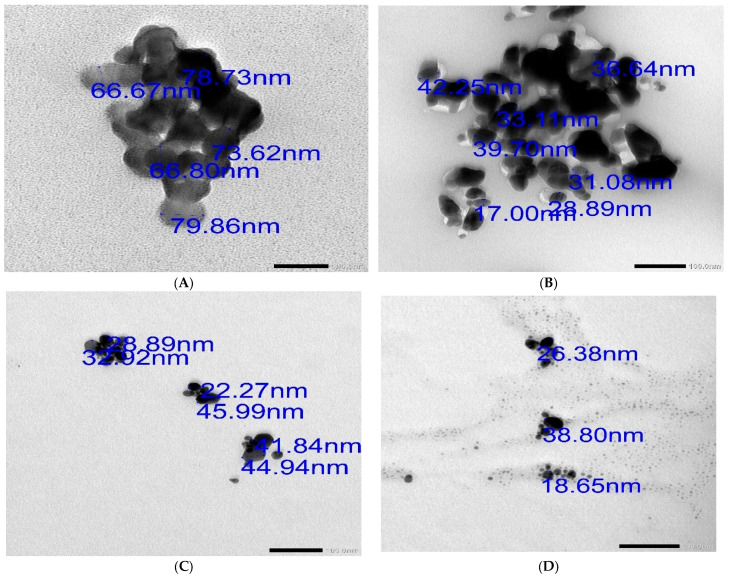
TEM: (**A**) NTZ Citrus Cs/Ag NPs. (**B**) Citrus Cs/Ag NPs. (**C**) NTZ Cs/Ag NPs. (**D**) Cs/Ag NPs.

TEM images of Ag NPs revealed that *C. paradisi* peel extract could be fabricated, and the Ag nanoparticles reduced/stabilized by chitosan are displayed in Figure 2. Particles are usually spherical in nature. The nanoparticles’ size distribution ranged from 17 to 80 nm. The majority of NPs were uniformly sized, with only a small number exceeding 60 nm. NTZ Citrus Cs/Ag NPs were larger than 60 nm and aggregated, as shown in Figure 2A. Citrus Cs/Ag NPs had a smaller aggregated spherical shape, as shown in Figure 2B. NTZ Cs/Ag NPs were smaller and dispersed, as shown in Figure 2C. Cs/Ag NPs had the smallest size, as seen in Figure 2D.

#### 3.2.4. Fourier Transform Infrared Spectroscopy (FTIR)

The FTIR spectra of NTZ Citrus Cs/Ag NPs have four strong absorption peaks. A broad band at 3435.91 cm^−1^ is caused by the O–H group of the overlapping stretching vibration due to phenolic compounds and the N–H stretching vibration of the NH_2_ group. The 2006.64 cm^−1^ peak aligned with CH and CH_2_ stretching vibrations. The carbonyl group (C=O) stretching mode was detected at 1534. 31 cm^−1^, indicating the presence of substances containing carboxylic, aldehydes, esters, or ketone groups derived from tannins and flavonoids. The green synthesized Ag NPs displayed a broad band fingerprint at 711 cm^−1^.

**Figure 3:**
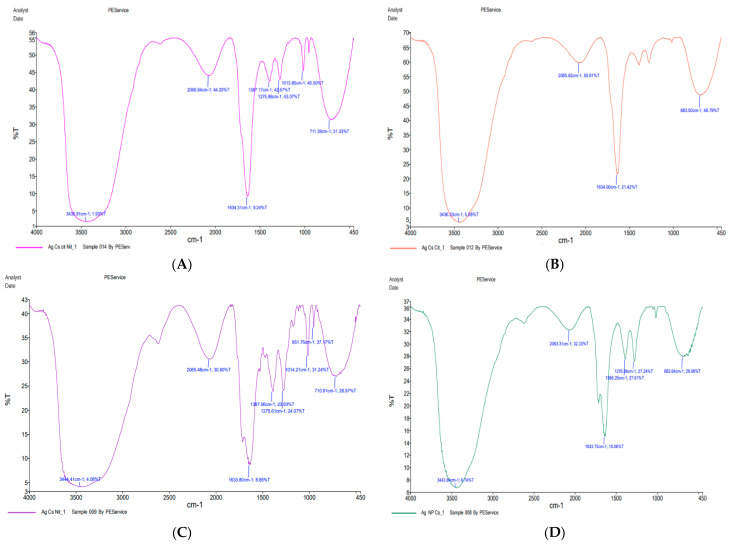
FTIR: (**A**) NTZ Citrus Cs/Ag NPs. (**B**) Citrus Cs/Ag NPs. (**C**) NTZ Cs/Ag NPs. (**D**) Cs/Ag NPs.

The peak at 2065.82 cm^−1^ correlates to stretching C-H bending vibration modes, the 1634 cm^−1^ peak represents C=O stretching and N–H bending primary amides, and the 683.5 cm^−1^ band resulted from bending vibrations, confirming the presence of the C-CL group. NTZ Cs/Ag NPs and Cs/Ag NPs showed these exact same peaks, with additional peaks at 1387.06, 1275.61, 1014.21, 951.75, and 710.51 cm^-1^ in NTZ Cs/Ag NPs and at 1386.2 and 1276 cm^−1^ in Cs/Ag NPs. The peak at 1387.06 and 1386.2 was observed in the presence of C–C and C–N stretching, the peak at 1275.61 and 1276 cm^−1^ was caused by C–O stretching (1210–1320 cm^−1^), the peak at 1014.21 cm^−1^ resulted from C–OH stretching alcohols or a C-stretching ether group, and the peak at 951.75 cm^–1^ contained functional groups, mainly from carbohydrates (Figure 3).

### 3.3. Oocyst Shedding

At the 20th day PI, which correlates with the experiment end, the diseased model group (GII) displayed the highest mean count of *Cryptosporidium* oocysts (481.2 ± 6.0), then came the infected mice that were treated with Cs/Ag NPs (GVI) (437.1 ± 1.7), with a high significant difference statistically between the two groups (*p* < 0.001). The mean count of *Cryptosporidium* oocysts in the treated groups (GIII-IX) is significantly lower than that in the infected non-treated control group (GII) (*p* < 0.001). Combining the Citrus Cs/Ag NPS and nitazoxanide displayed the best efficacy (96%), with the lowest mean count of *Cryptosporidium* oocysts (20.8 ± 1.9). The efficacy of Citrus alone (79%) was higher than nitazoxanide alone (61%), with this difference being statistically significant (*p* < 0.001), whereas there was no significant difference when compared to nitazoxanide loaded on Cs/Ag NPs (*p* = 0.8). Utilizing Citrus in the synthesis of Cs/Ag NPs and loading nitazoxanide on Cs/Ag NPs improved their efficacy, reaching 91% and 78%, respectively (Table 1).

Table 1 shows the *Cryptosporidium* oocyst number per gram stool as (mean ± SE) × 10^3^. A percentage representing the inhibition is given using this formula: reduction percentage = [(oocyst mean count in the infected control group − oocyst mean count in the infected treated group)/oocyst mean count in the infected control group] × 100. The mean values in each row are significantly different from those with different superscript small letters, but those with the same one are similar. The superscript capital letters in each column indicate whether the mean values are similar via the same superscript capital letters or significantly different via different superscript capital letters (PI stands for post-infection, and PR stands for reduction percent).

### 3.4. Histopathological Findings

**Figure 4:**
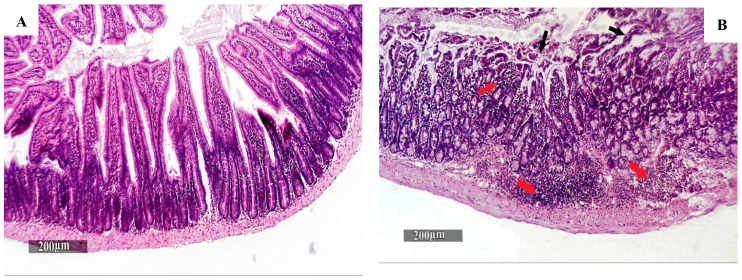

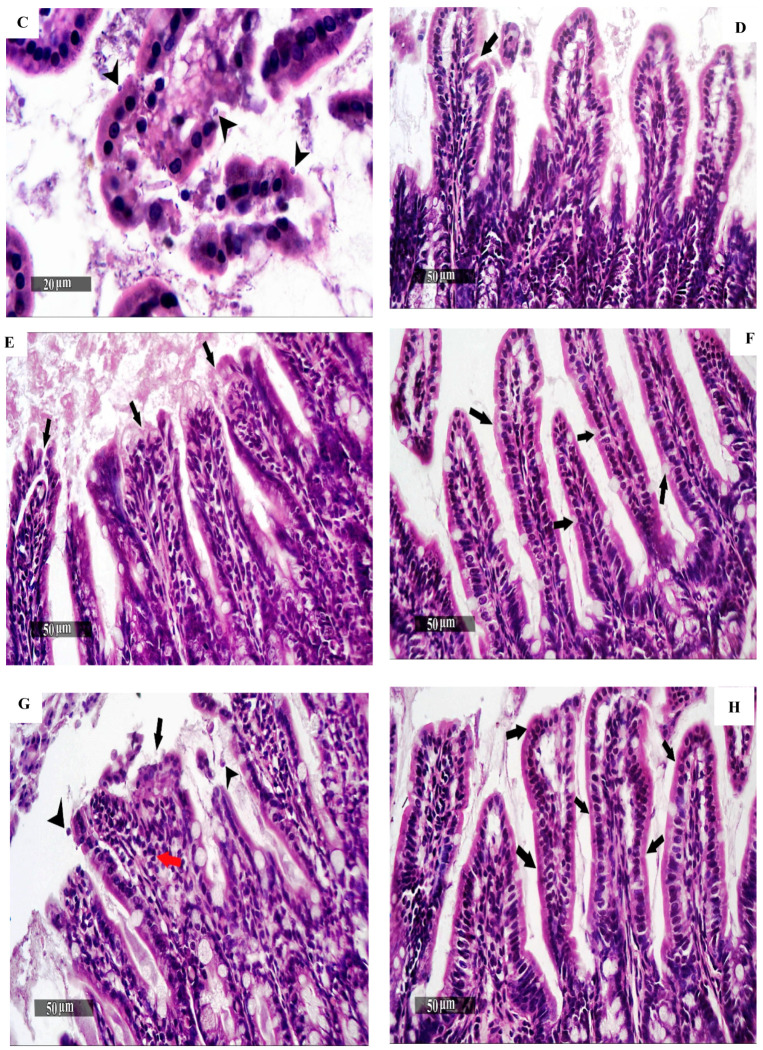

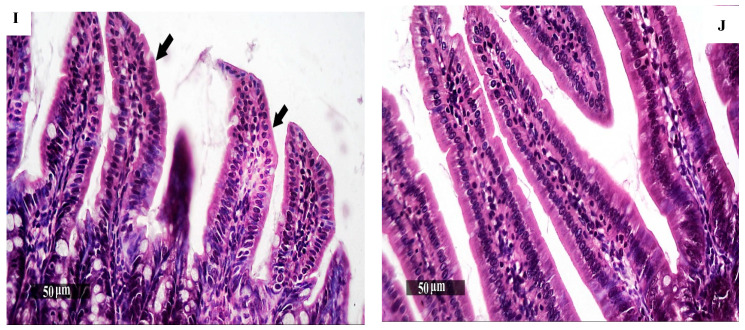
H & E-stained intestinal sections from various groups. (**A**) **GI** displays a normal intestinal architecture with intact intestinal mucosa, including apparent intact villi, intestinal crypts with an intact lining epithelium and goblet cells, and intact lamina propria, as well as an intact brush border. (**B**) **GII** shows multiple focal disorganizations and fragmentations of the intestinal villi tips covering the epithelium (black arrows) with a remarkably higher number of mononuclear inflammatory cells infiltrating the lamina propria, as well as submucosal layers (red arrows). (**C**) Adherent *Cryptosporidium* oocysts (arrowheads). (**D**) **GIII** demonstrates a moderate improvement in intestinal wall morphologies, with some evidence of intestinal villi degenerative changes or fragmentation (black arrow). (**E**) **GIV** shows partial villous blunting, with focal ulceration of the intestinal mucosa (black arrows). (**F**) **GV** shows well-organized histological features in the intestinal wall, with almost apparent intact intestinal villi and intestinal crypts (black arrows). (**G**) **GVI** shows fragmentation in the intestinal villi tips covering the epithelium (black arrow) with many adherent oocysts (arrowheads) and higher mononuclear inflammatory cells infiltrating the lamina propria (red arrow). (**H**) **GVII** shows higher protective efficacy, with more enhanced morphological features in the intestinal wall and intestinal villi covering the entire epithelium (black arrows) without abnormal alteration evidence. (**I**) **GVIII** demonstrates moderate improvements in intestinal wall morphologies, with some evidence of intestinal villi degenerative changes or fragmentation (black arrows). (**J**) **GIX** demonstrates the best re-organized and enhanced histological features among the treatment groups (resembling healthy models).

The histopathological analysis of intestinal sections stained with H&E from different study groups is displayed in Figure 4. Sections from the healthy model (GI) display typical organized morphological structures in the intestinal wall, with intact intestinal mucosa, including apparent intact villi, intestinal crypts with intact lining epithelium and goblet cells, and intact lamina propria, as well as an intact brush border (Figure 4A). Sections from the diseased model (GII) show multiple instances of focal disorganization and fragmentation in the intestinal villi tips covering the epithelium and a remarkably higher number of mononuclear inflammatory cells infiltrating the lamina propria, as well as submucosal layers with many instances of border-adherent oocysts of *Cryptosporidium*, shown as oval or rounded purple-stained bodies, with a diameter ranging from 4 to 6 µm (Figure 4B,C).

Examination of the different treated groups’ intestinal sections (Figure 4D–J) revealed significant improvement in intestinal wall morphologies and minimal evidence of intestinal villi degenerative changes, fragmentation, and intestinal inflammation, with the group treated with NTZ Citrus Cs/Ag NPs (GIX) (Figure 4J) displaying the best results. No remarkable difference is detected between the diseased model (GII) and the infected group treated with Cs/Ag NPs (GVI) (Figure 4G).

### 3.5. Immunohistochemical Studies

**Figure 5:**
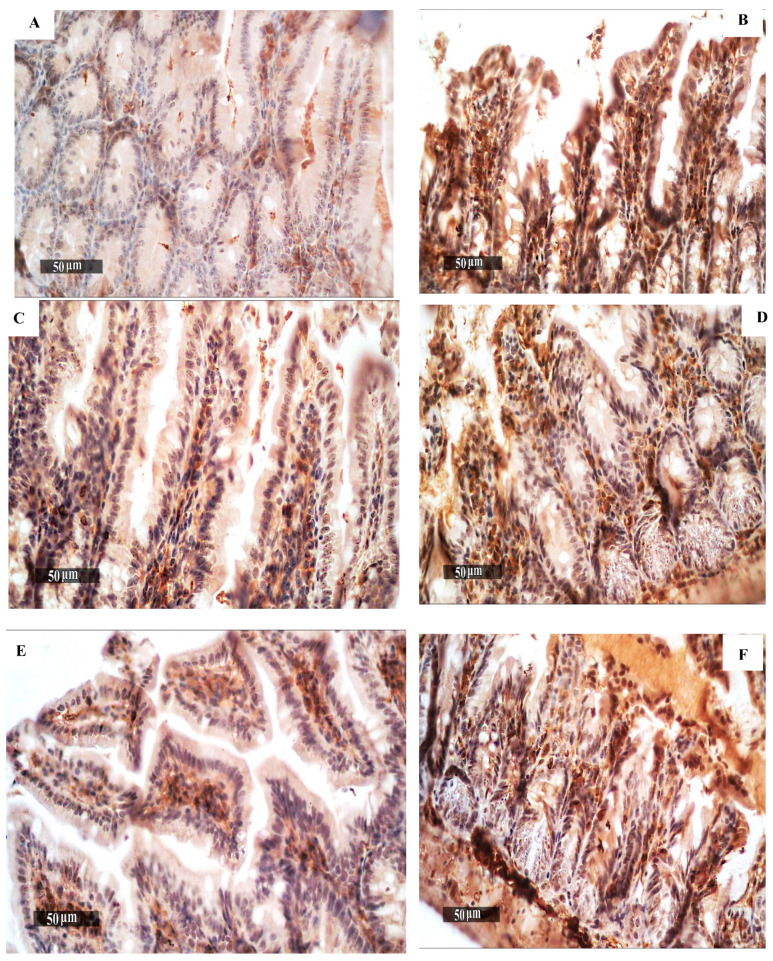

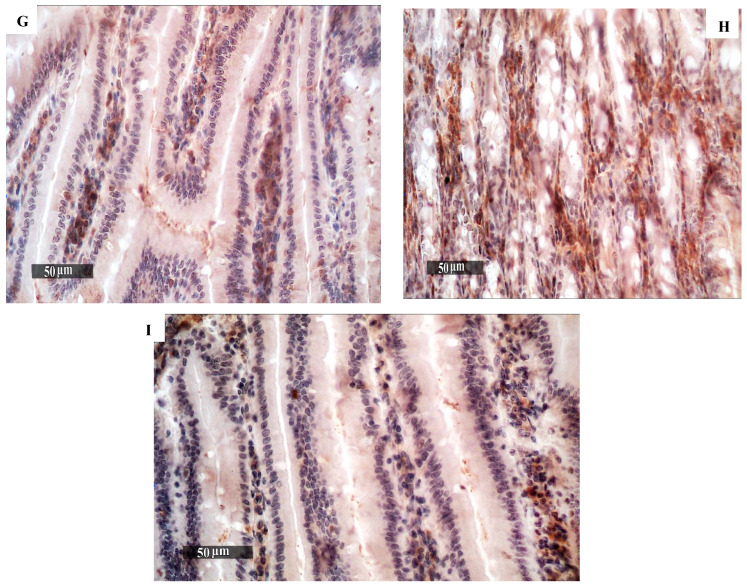
NFκB immunohistochemical expression in the mouse intestine, (**A**) Group I (the healthy model) with reduced NFκB expression. (**B**) Group II showed increased NFκB levels. (**C**–**I**) Treated groups demonstrated NFκB expression reductions.

Quantitative examination of sections from the small intestine demonstrated a significant increase in NFκB expression in group II (the diseased model) samples (Figure 5B) compared with group I (the healthy model) samples (Figure 5A). A significant decrease in NFκB expression was documented in the treated groups III–IX (Figure 5C–I) compared to group II (Figure 5B). The group treated with NTZ Citrus Cs/Ag NPs (GIX) displayed the highest reduction (Table 2).

### 3.6. Immunological Analysis of Serum Levels of TNF Alpha and Interleukin 10 Cytokines

**Figure 6:**
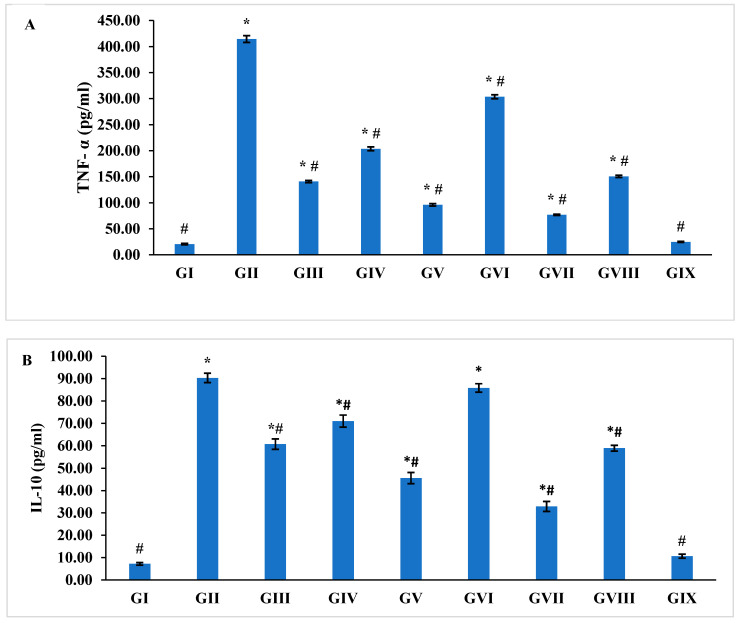
The serum levels of (**A**) Tumor Necrosis factor (TNF-α) and (**B**) Interleukin 10 (IL-10) in different study groups (data are presented as mean ± SE. * *p* < 0.05 versus normal model group, # *p* < 0.05 versus diseased model group.

Regarding the levels of TNF-α and Il-10, mice in the diseased model group (GII) showed significantly (*p* < 0.005) increased levels (414.4 ± 6.5, 90.3 ± 2.1, respectively) compared with the healthy model group (GI) (20.6 ± 1.3, 7.2 ± 0.6, respectively). The TNF-α and Il-10 levels decreased significantly (*p* < 0.001) in the group treated with NTZ Citrus Cs/Ag NPs (GIX), showing the best results (24.8 ± 0.9) with a non-significant difference from the healthy model group (GI) (*p* = 0.9) (Figure 6).

## 4. Discussion

Immunocompromised patients are thought to be at high risk for contracting a *cryptosporidium* infection [33]. Ref. [34] states that no effective therapy or vaccination is available for cryptosporidiosis. Therefore, biosynthesized silver nanoparticles (Ag NPs) can be utilized as a novel option for treating *C. parvum* infection via releasing silver ions (Ag+), which induce oxidative stress by releasing reactive oxygen species. Both the sporozoites and the oocyst cell wall can be destroyed by the discharged Ag+ ions and nanoparticles [35].

Novel nanocomposites with synergistic capabilities can be developed by combining biopolymers of various functionalities with metal nanoparticles. Additionally, loading chitosan with various metals enhances the chitosan’s antimicrobial properties. For a range of commercial applications, chitosan-based silver nanocomposites that combine chitosan with silver nanoparticles are being produced [27].

To improve the interpretation of data, nanoparticles had to be physicochemically characterized. To offer clear insights into the crystalline nature, chemical composition, surface property morphology, and particle size of chemically and green-synthesized Ag NPs, a combination of UV-Vis, FT-IR, and TEM analysis was used in the nanoparticle characterization method. Silver nanoparticle synthesis is indicated by the color shift, which is corroborated by the characteristic surface plasmon resonance (SPR) peak appearance between 400 and 500 nm [36]. The 430–450 nm peaks that were seen in this study verified the formation of silver nanoparticles. Several factors, encompassing the volume of plant extract, temperature, pH, duration, and concentration of silver nitrate, influence the production of Ag NPs.

The phytoconstituent of *Citrus paradisi* peel extract contains several functional groups identified by FTIR spectroscopy as being in charge of the bioreduction of Ag+ ions and the further capping and stabilization of Ag NPs. To determine the pertinent functional groups, the measured bands, and peaks were compared to standard values [37]. The FTIR spectrum obtained indicated that the surface of the synthesized Ag NPs included a variety of functional groups, alcohol, alkenes, alkynes, carboxylic, amine, esters, aldehydes, or ketone-containing compounds derived from tannins and flavonoids. The FT-IR spectrum analysis of all the observed data was consistent with earlier studies. The findings of [38] and [39] suggest that the synthesized Ag NPs include the indicated phytochemicals, as evidenced by the spectrum pattern similarities between the plant fruit extract and the synthesized Ag NPs. The extract may have shown capping and reducing capabilities due to these compositions and functional groups.

The biosynthesized Ag NPs were shown to be uniform nanoparticles in the 17–80 nm range in TEM images. Most NPs were uniformly sized, with just a small number exceeding 60 nm. The Ag NPs produced by the extract of *Malus domestica* have a spherical shape, polydispersity, and a size range of 40 to 100 nm, according to the study by [40]. Additionally, Ag NPs with a size range of 10 to 60 nm were synthesized from *Origanum majorana* by Yassin et al. (2022), demonstrating polydispersity.

The green-synthesized Cs/Ag NPs significantly reduced the *C. parvum* oocyst count in the current study. Compared to the diseased model group, the treated groups had a significantly lower count of *Cryptosporidium* oocysts. Interestingly, the combination of nitazoxanide and Citrus Cs/Ag NPs showed higher activities and produced the maximum effectiveness (96%) compared to each of them alone. These results agree with those of [27], who documented that the lemon juice-extract-mediated biosynthesis of silver nanoparticles with chitosan showed remarkable antioxidant and antibacterial properties.

Furthermore, the histopathological analysis of intestinal sections from various treatment groups showed a notable improvement in the morphologies of the intestinal walls and a reduction in the number of intestinal villi degenerative changes, fragmentation, and inflammation. The group that received treatment with a combination of nitazoxanide Citrus Cs/Ag NPs showed the best results. No significant differences were found between the infected group that was given blank nanoparticles and the infected group that was not treated. These results were consistent with those of other researchers who observed that the intestinal histopathological sections had improved in mice that received combined NTZ-artesunate loaded on polymeric nanofiber and NTZ-loaded chitosan NPs, respectively. These researchers also detected minimal *Cryptosporidium* oocysts with mild inflammatory cellular infiltration at the intestinal epithelium.

In addition to being an essential component of several physiological and pathological processes, NF-κB is a potent immune response orchestrator and plays a crucial role in modulating responses to various external stimuli [41]. NF-κB’s function during certain parasite infections has been the subject of several studies in the literature. The nuclear factor kappa B (NF-κB) is activated by *C. parvum* within hours of infection, which triggers the anti-apoptotic processes [42]. To identify NF-κB as a marker for T helper cells, cytotoxic T cells, apoptosis, and inflammation in the groups with the healthy model, diseased model, and infected–treated model, we performed immunohistochemistry quantitative evaluation of a small intestine section. When comparing the infected group to the healthy control group, the immunohistochemical findings confirmed a significant increase in NF-κB expression. However, the NF-κB expression in the treated groups was significantly lower than in the infected group. The group showed a significant decrease when nitazoxanide and Citrus Cs/Ag NPs were administered together.

To reduce pro-inflammatory cytokines and enhance anti-inflammatory cytokines, various medicinal plants, and medications have been explored for *C. parvum* infections, mainly through immune response regulation [43]. Compared to the control group, the infected group had significantly higher levels of TNF-α and Il-10 (*p* < 0.001). The combination of nitazoxanide and Citrus Cs/Ag NPs showed the best outcomes, with no significant difference from the healthy model group. The TNF-α and Il-10 levels were reduced significantly in the treatment groups (*p* < 0.001) compared to the diseased model group.

## 5. Conclusions

The current study documented that utilizing *C. paradisi* extract in the green synthesis of Cs/Ag NPs has a potential role as an anti-cryptosporidiosis therapeutic agent. The combination of nitazoxanide and Citrus Cs/Ag NPs demonstrated the best results against infection and could be considered an alternative treatment for *C. parvum*. Treatment based on nanocomposites has a vital role in overcoming cryptosporidiosis, which requires further studies to elucidate the mechanisms involved.

## Figures and Tables

**Table 1:** Count of *Cryptosporidium* oocysts per gram stool (×10^3^) in different groups.

Group	7th Day PI	10th Day PI		12th Day PI		15th Day PI		20th Day PI	
	Mean ± SE	Mean ± SE	PR%	Mean ± SE	PR%	Mean ± SE	PR%	Mean ± SE	PR%
**GII**	475.6 ± 4.6 ^aA^	477.9 ± 5.9 ^aA^	-	478.9 ± 5.8 ^aA^	-	480.1 ± 5.9 ^aA^	-	481.2 ± 6.0 ^aA^	-
**GIII**	477.9 ± 4.0 ^aA^	108.2 ± 2.1 ^bB^	77%	103 ± 1.7 ^bB^	79%	102.3 ±1.4 ^bB^	79%	100.9 ± 1.2 ^bB^	79%
**GIV**	479.4 ± 2.5 ^aA^	197.9 ± 3.4 ^bC^	59%	189.1 ± 3.6 ^bC^	61%	189 ± 3.2 ^bC^	61%	186 ± 2.0 ^bC^	61%
**GV**	475.8 ± 4.2 ^aA^	81.7 ± 1.6 ^bD^	83%	73.7 ± 1.9 ^bD^	85%	73.6 ± 1.7 ^bD^	85%	73.1 ± 1.9 ^bD^	85%
**GVI**	477.1 ± 4.3 ^aA^	443.6 ± 1.9 ^bE^	7%	440 ± 1.7 ^bE^	8%	438.9 ± 1.0 ^bE^	9%	437.1 ± 1.7 ^bE^	9%
**GVII**	476.7 ± 5.9 ^aA^	51.1 ± 1.5 ^bF^	89%	49.5 ± 2.1 ^bF^	90%	48.2 ± 1.1 ^bF^	90%	44 ± 1.2 ^bF^	91%
**GVIII**	478.2 ± 4.6 ^aA^	118 ± 3.1 ^bB^	75%	112.3 ± 2.2 ^bB^	77%	111.9 ± 3.3 ^bB^	77%	107.6 ± 4.4 ^bB^	78%
**GIX**	475.6 ± 3.3 ^aA^	30.4 ± 2.5 ^bG^	94%	26.5 ± 2.0 ^bG^	95%	25.5 ± 1.5 ^bG^	95%	20.8 ± 1.9 ^bG^	96%

**Table 2:** The percentage of P-NFkB immunohistochemical expression in different study groups.

Groups	Area % of Immunohistochemical Expression of NFkB	
	Mean ± SE	ANOVA *p*-Value
**GI**	2.9 ± 0.2	0.001 **
**GII**	34.2 ± 1.3	
**GIII**	8.7 ± 0.4	
**GIV**	17 ± 1	
**GV**	3.8 ± 0.2	
**GVI**	19 ± 1	
**GVII**	3.5 ± 0.2	
**GVIII**	10 ± 0.7	
**GIX**	3 ± 0.5	

** *p*-Value < 0.01 is highly significant.

## Data Availability

All data generated or analyzed during this study are included in this published article.

## Supplementary Materials

The following are available online at https://www.mdpi.com/article/10.3390/pharmaceutics16070968/s1, Figure S1: displays visual observation of A: Ag NPs, Ag No3. B: Cs/Ag NPs. C: Citrus Cs/Ag NPs. D: NTZ Cs/Ag NPs. E: NTZ Citrus Cs/Ag NPs.
