# Supplementary material for: Green Synthesis of Chitosan/Silver Nanoparticles Using Citrus paradisi Extract and Its Potential Anti-Cryptosporidiosis Effect
Source: Pharmaceutics. 2024 Jul 22;16(7):968. doi: 10.3390/pharmaceutics16070968 (PMC11279850; doi:10.3390/pharmaceutics16070968)
Supplement: Supplementary file 1 [file pharmaceutics-16-00968-s001.zip › pharmaceutics-3093028-supplementary.pdf]

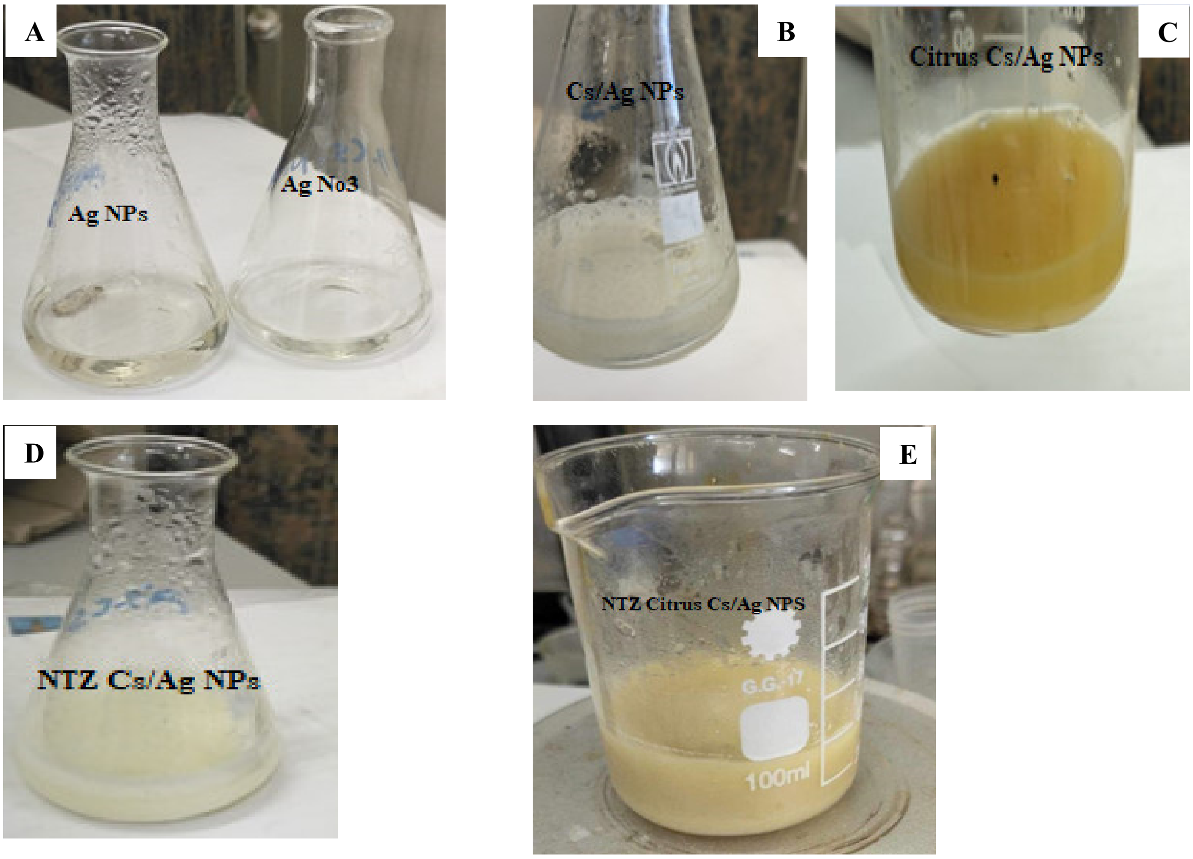

Figure S1: displays visual observation of A: Ag NPs, Ag No3. B: Cs/Ag NPs. C: Citrus Cs/Ag NPs. D: NTZ Cs/Ag NPs. E: NTZ Citrus Cs/Ag NPs.
